# Increased α-actinin-1 destabilizes E-cadherin-based adhesions and associates with poor prognosis in basal-like breast cancer

**DOI:** 10.1371/journal.pone.0196986

**Published:** 2018-05-09

**Authors:** Bianca Kovac, Tomi P. Mäkelä, Tea Vallenius

**Affiliations:** Research Programs Unit, Faculty of Medicine and Helsinki Institute of Life Science HiLIFE, University of Helsinki, Biomedicum Helsinki, Helsinki, Finland; University of South Alabama Mitchell Cancer Institute, UNITED STATES

## Abstract

The controlled formation and stabilization of E-cadherin-based adhesions is vital for epithelial integrity. This requires co-operation between the E-cadherin-based adhesions and the associated actin cytoskeleton. In cancer, this co-operation often fails, predisposing cells to migration through molecular mechanisms that have only been partially characterized. Here, we demonstrate that the actin filament cross-linker α-actinin-1 is frequently increased in human breast cancer. In mammary epithelial cells, the increased α-actinin-1 levels promote cell migration and induce disorganized acini-like structures in Matrigel. This is accompanied by a major reorganization of the actin cytoskeleton and the associated E-cadherin-based adhesions. Increased expression of α-actinin-1 is particularly noted in basal-like breast cancer cell lines, and in breast cancer patients it associates with poor prognosis in basal-like subtypes. Downregulation of α-actinin-1 in E-cadherin expressing basal-like breast cancer cells demonstrate that α-actinin-1-assembled actin fibers destabilize E-cadherin-based adhesions. Taken together, these results indicate that increased α-actinin-1 expression destabilizes E-cadherin-based adhesions, which is likely to promote the migratory potential of breast cancer cells. Furthermore, our results identify α-actinin-1 as a candidate prognostic biomarker in basal-like breast cancer.

## Introduction

The dynamic actin cytoskeleton co-operates with E-cadherin- and integrin-based cell-cell or cell-matrix adhesions to maintain polarized epithelial organization and to generate the force required for cell shape changes and cell migration in remodeling tissues [1]. In malignant epithelia, the controlled co-operation between actin and adhesions often fails, resulting in the loss of polarized epithelial organization and increased morphological cell plasticity that predisposes cancer cells to invade and disseminate [2–4]. According to a traditional view cancer cells invade and disseminate from primary tumors as single cells through epithelial to mesenchymal transition (EMT) [4, 5]. However, recent evidence suggests that cells with intermediate or partial EMT can also migrate collectively, i.e. as group of clustered cells [2, 6–8]. This is termed collective invasion. Collective invasion is associated with the expression of E-cadherin at cell-cell adhesions [9–11]. To acquire the morphological plasticity required for cell migration, the migrating cancer cell clusters need to destabilize E-cadherin-based adhesions between adjacent cells. Previous cell culture and model organism studies have reported that the actin cytoskeleton is required for the destabilization process. This has been demonstrated in cultured cells through traditional Ca^2+^ switch experiments [12] or by altering myosin II activity [13]. Cell intercalation during *Drosophila* germband extension is an *in vivo* example of dynamic co-operation between the actin cytoskeleton and E-cadherin-based adhesions [14]. While the requirement of the actin cytoskeleton in stabilizing E-cadherin-based adhesions is clear, the actin-related stability regulators involved have only been partially characterized.

Of the possible actin-related regulators in the context of cancer, the major filamentous actin (F-actin) cross-linkers non-muscle α-actinin-1 and α-actinin-4 are of interest. Numerous studies have shown that the upregulation of α-actinin-4 is associated with a poor prognosis in various cancers, including breast, colorectal, ovarian, pancreatic and salivary gland cancers [15–19]. Both loss- and gain-of-function approaches have demonstrated that the increased α-actinin-4 levels promote cancer cell migration and invasion [16, 20–23]. Furthermore, it is thought that the increased α-actinin-4 levels contribute to the EMT process. For example, in colon cancer biopsies, high α-actinin-4 staining has been detected at the invasive cancer front, where cells lack E-cadherin expression [16]. Other studies suggest that the repression of E-cadherin expression can occur by upregulating Snail [20] or through interaction between α-actinin-4 and β-catenin in the absence of E-cadherin [21].

While α-actinin-4 and α-actinin-1 are widely expressed, closely related genes that have overlapping functions [24, 25], the association of α-actinin-1 expression in cancer is largely uncharacterized [26–28]. In the present study, we determine that α-actinin-1 expression is frequently increased in human breast cancer and is associated with the poor prognosis of basal-like breast cancer. In breast cancer cells, increased α-actinin-1 levels result in the destabilization of E-cadherin-based adhesions, which can promote the migratory potential of collectively migrating cancer cells.

## Results

### α-actinin-1 expression is frequently increased in human breast cancers

**Fig 1:**
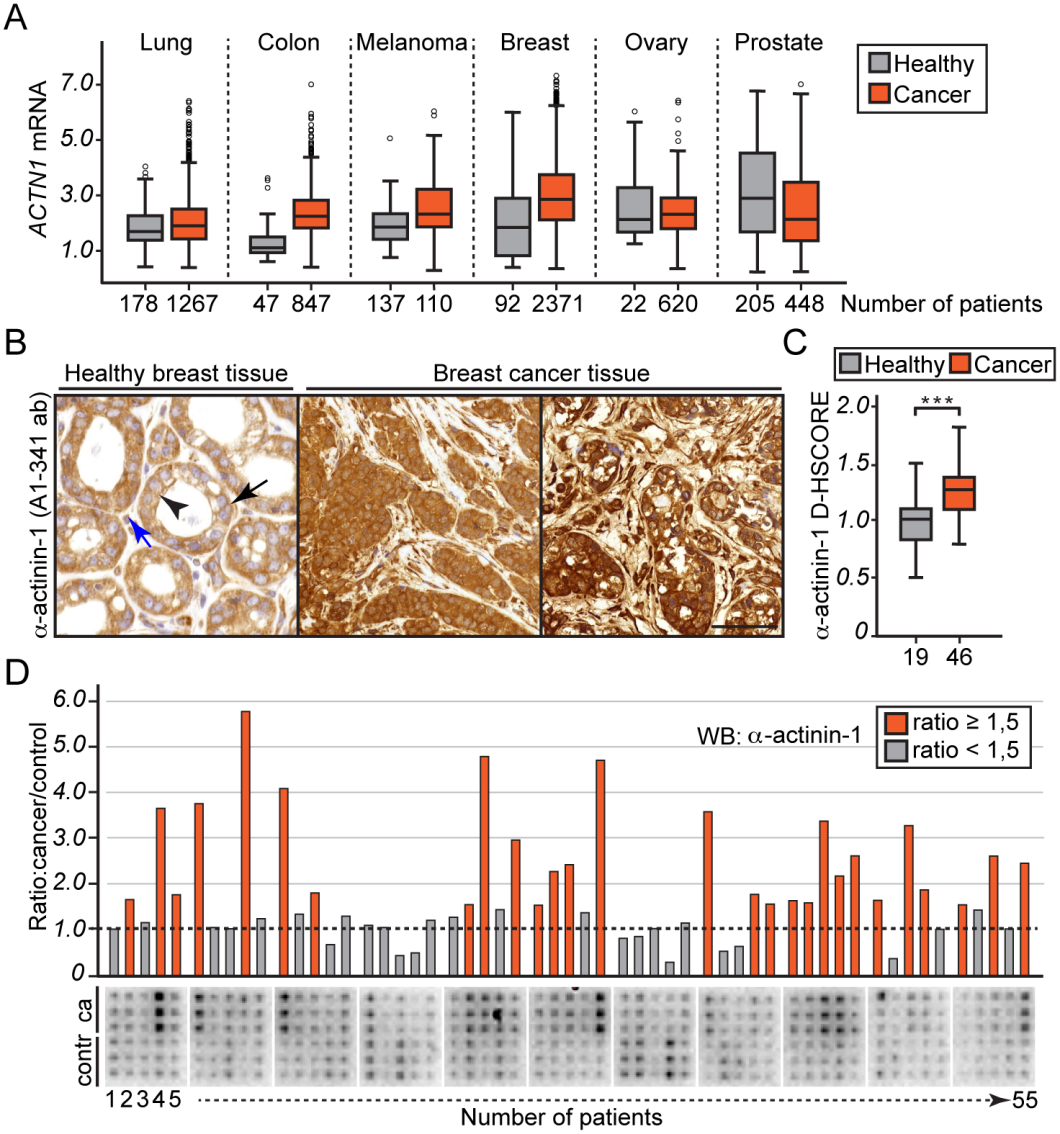
α-actinin-1 expression is increased in human breast cancer. (A) Comparison of *ACTN1* mRNA in healthy (grey boxes) and cancerous tissues (red boxes) as indicated. Box plots show median expression as a line, with 25 and 75 percentiles as lower and upper boxes with whiskers and outlier points extending to cover remaining data [29]. The x-axis indicates the number of patients. (B) Representative immunohistochemistry images of a healthy and two breast cancer sections stained for α-actinin-1 (A1-341 ab). The black arrowhead in healthy section indicates a luminal cell, and a black arrow indicates a myoepithelial cell. The blue arrow in the same image shows a stromal fibroblast positive for α-actinin-1. Scale bar, 50 μm. (C) D-HSCORE score [33] to compare α-actinin-1 staining signal of healthy (n = 19) and breast cancer tissues (n = 46) stained in (B). ***P<0.001 by student’s t-test. (D) Human breast cancer tissue lysate array immunoblotted for α-actinin-1. Each vertical line of spots represents triplicates of cancer (ca) and matched healthy (contr) tissues from a patient (n = 55). The columns represent the expression ratio of α-actinin-1 between cancer and adjacent healthy tissue of each patient based on the average spot intensity of triplicates. The red columns indicate ≥1.5-fold increase in α-actinin-1 levels.

To compare the expression of α-actinin-1 in healthy and cancer tissues, we performed *in silico* transcriptomic analysis [29]. Consistent with previous reports [30, 31], α-actinin-1 mRNA is widely expressed in healthy tissues (Fig 1A, grey boxes). Interestingly, the comparison of healthy and cancer tissues revealed that in a portion of cancers, such as colon, melanoma and breast, α-actinin-1 levels are increased (Fig 1A, red boxes). Motivated by this observation, we selected to study the role of α-actinin-1 in breast cancer. To evaluate α-actinin-1 expression and distribution in breast tissue, we performed immunohistochemistry analysis on paraffin-embedded breast tissue microarray slides containing both healthy and cancer lesions using a rabbit polyclonal α-actinin-1 antibody (A1-341) [32]. We confirmed the specificity of the antibody using α-actinin-1 deficient cells and antibody blocking approaches (S1A–S1C Fig). In healthy breast tissue, the α-actinin-1 antibody stains both luminal (Fig 1B, black arrowhead) and myoepithelial cells (Fig 1B, black arrow). In both cell types, the signal is primarily cytoplasmic and extends to the cell-cell and cell-matrix borders. In addition to epithelia, cytoplasmic α-actinin-1 staining is apparent in stromal fibroblasts (Fig 1B, blue). Cancerous tissues revealed intense, cytoplasmic α-actinin-1 signal (Fig 1B, Breast cancer tissue). Additionally, we observed a weak nuclear signal in a portion of the samples. To assess whether α-actinin-1 staining was increased in cancer lesions compared to healthy controls, we used the digital H-score method (D-HSCORE [33]) (S1D Fig). This analysis revealed a 1.3-fold increase in α-actinin-1 staining in cancer tissues (n = 46) compared to healthy tissues (n = 19, p-value 2.3x10^-4^) (Fig 1C). Finally, we performed western blotting analysis of a breast cancer tissue lysate microarray containing 55 breast cancer patient samples and adjacent healthy tissues in triplicates using the A1-341 α-actinin-1 antibody (Fig 1D). The quantification of paired cancer and healthy tissues indicate that 51% of cancer samples show over a 1.5-fold increase in α-actinin-1 levels (*p*-value 7.5x10^-5^). Taken together, the mRNA profiling, immunohistochemistry and immunoblotting analyses demonstrate that α-actinin-1 levels are frequently increased in human breast cancer. Therefore, α-actinin-1 may have a role in breast cancer progression.

### Increased α-actinin-1 promotes cell migration and a loss of polarity by reorganizing the actin cytoskeleton and E-cadherin-based adhesions

**Fig 2:**
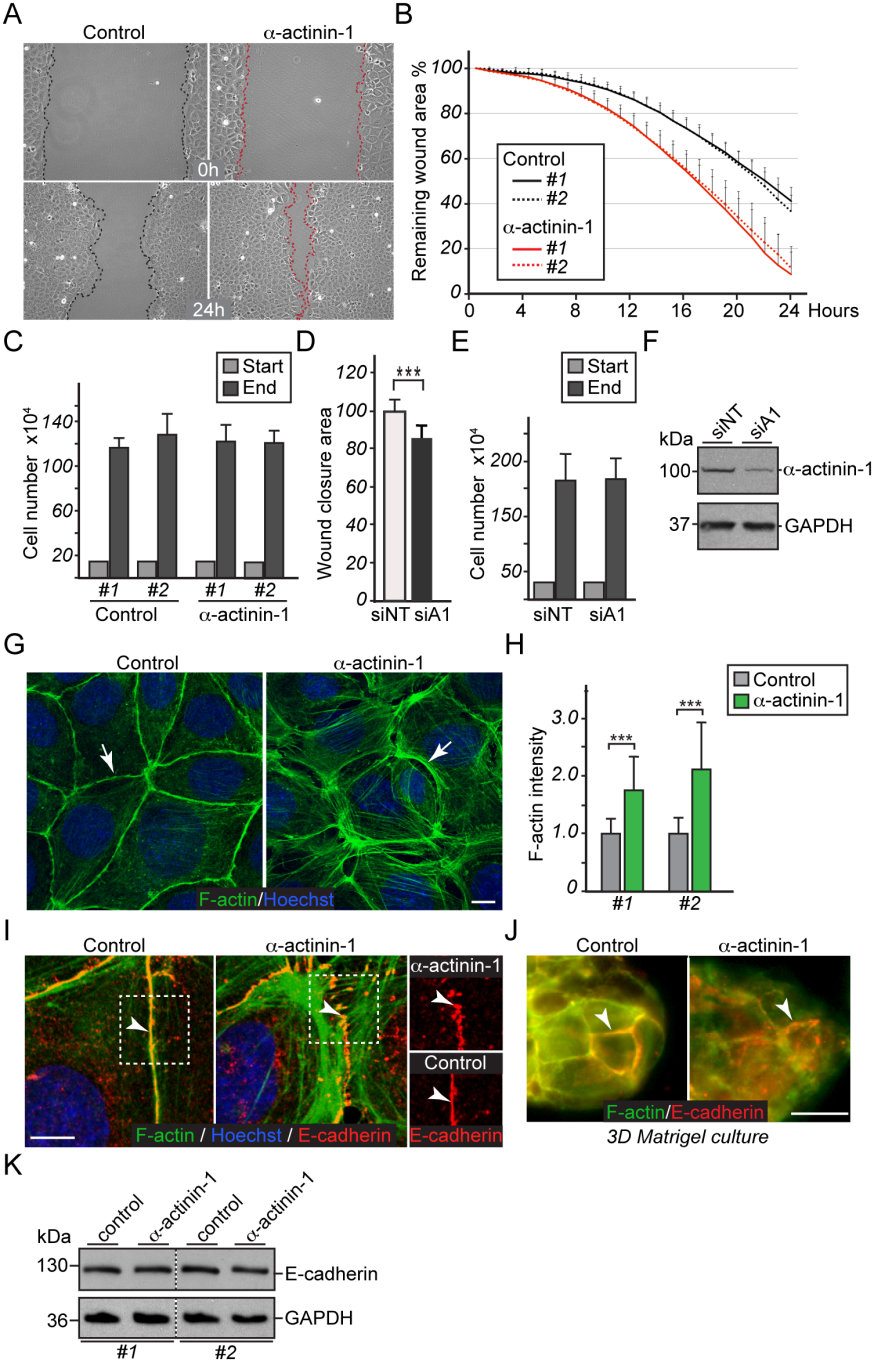
Increased α-actinin-1 promotes cell migration and reorganizes F-actin and E-cadherin based adhesions. (A-C) EpH4 mammary epithelial cells stably overexpressing control (Control) or α-actinin-1 (α-actinin-1). (A) Phase-contrast images of scratch wounds of control and α-actinin-1 expressing cells at the time of scratch (0 h) and 24 h. (B) Quantification of the remaining wound area (n = 9 wounds/line #) from two independent experiments. (C) Cell number of control (#1, #2) and α-actinin-1 (#1, #2) expressing EpH4 cells seeded for wound healing (Start, light grey) and at end point (End, dark grey). Error bars indicate s.d. of triplicates from two independent experiments. No statistical difference by Student’s t-test between cell lines. (D-F) Wound healing assay in human HCC1937 breast cancer cells following siRNA mediated downregulation of control (siNT, n = 20) or α-actinin-1 (siA1, n = 20) expression from two independent experiments. (D) Remaining wound closure area. (E) Cell number seeded for wound healing (Start, light grey) and at 20 h end point (End, dark grey). Error bars indicate s.d. from two independent experiments. No statistical difference by Student’s t-test between downregulated cells. (F) Western blotting analysis with α-actinin-1 antibody to show downregulation efficiency in HCC1937 cells. (G) Confocal immunofluorescence images of phalloidin (F-actin, green) and Hoechst (blue) stained cells. Arrows indicate F-actin reorganization. Scale bar, 10 μm. (H) Quantification (n = 42-52/line #) of F-actin intensity shown in (G) from two independent experiments. Values are normalized to control cells and represent an arbitrary value. (I) Merged immunofluorescence images of phalloidin (F-actin, green), E-cadherin (red) and Hoechst (blue), and on the right, E-cadherin alone. Arrowheads indicate the punctate E-cadherin pattern. Scale bar, 10 μm. (J) Phalloidin (F-actin, green) and E-cadherin (red) co-staining of control and α-actinin-1 expressing cells grown on three-dimensional Matrigel gel for seven days. Arrowheads indicate the E-cadherin pattern. Scale bar, 20 μm. (K) Western blotting analysis with E-cadherin and GAPDH antibodies to demonstrate unaltered E-cadherin levels. Error bars indicate s.d. ***P<0.001 by Student’s t-test.

To address whether the increased α-actinin-1 expression in mammary epithelial cells can cause functional alterations that are relevant for cancer progression, we generated stable EpH4 mammary epithelial cell lines expressing GFP alone (control) or GFP-tagged human α-actinin-1 (α-actinin-1) (S2A and S2B Fig). First, two control and α-actinin-1 lines were subjected to wound healing assays or embedded in Matrigel to follow possible functional alterations in the three-dimensional environment. We performed 24-hour time-lapse imaging every hour after scratch wounding and found that α-actinin-1-expressing cells collectively migrate in a more dynamic manner than control cells (Fig 2A and S1 Movie). Furthermore, α-actinin-1-expressing cells closed their wounds significantly faster than control cells (at the 24-hour time point, 25.7% difference, p-value 1.4x10^-6^) (Fig 2B), whereas cell number between control and α-actinin-1-expressing cells did not show significant difference (Fig 2C). Comparison of α-actinin-1-expressing and control cells growing on Matrigel for seven to ten days showed that both could form acini-like structures at same rate (S2C Fig). However, the structures that formed with α-actinin-1-expressing cells are larger and irregular in shape compared to control cells (S2D Fig, area and circularity), giving the impression of a disorganized structure. In support of the disorganization, α-actinin-1-expressing cells lack polarized laminin distribution, which is present in control cells (S2E Fig). In addition to overexpression approach, we performed siRNA-mediated downregulation of control (siNT) or α-actinin-1 (siA1) in human breast cancer cells, HCC1937, and subjected them to wound healing assay (Fig 2D–2F). Consistent with the overexpression result, α-actinin-1-downregulated cells closed their wounds significantly slower compared to control cells (at the 20-hour time point, 14% difference, p-value 1.1x10^-9^).

The increased cell migration and loss of polarity are cellular functions relevant in cancer progression [3]. To gain further insight into the cellular mechanisms involved, we stained cells for filamentous actin (F-actin), which revealed a major reorganization of the actin cytoskeleton in α-actinin-1 expressing cells (Fig 2G). Unlike to control cells, which have continuous linear F-actin staining between adjacent epithelial cells, α-actinin-1 expressing cells have frequent tiny gaps at cell-cell contacts and abundant subcortical actin fibers (Fig 2G, arrows). Quantification of F-actin intensity revealed an average 1.9-fold increase in F-actin intensity in α-actinin-1 expressing cells compared to control cells (Fig 2H, p-value 1.2x10^-11^). The F-actin phenotype prompted us to stain cells for E-cadherin. Interestingly, the E-cadherin signal is present at cell-cell adhesions in both control and α-actinin-1 expressing cells, but its distribution is distinct. While control cells have a continuous linear E-cadherin signal, α-actinin-1 expressing cells have a punctate E-cadherin staining pattern (Fig 2I, arrowheads in merged and E-cadherin-alone images). This difference in E-cadherin staining is also apparent in three-dimensional cell cultures (Fig 2J, arrowheads). Closer observation of F-actin and E-cadherin signals demonstrates that E-cadherin puncta are associated with short, perpendicular actin fibers (radial fibers) that are connected to the subcortical longitudinal actin fiber network (arc-like fibers) (Fig 2I, arrows). Subsequent immunoblotting for E-cadherin showed that control and α-actinin-1-expressing cells express comparable levels of E-cadherin (Fig 2K), arguing against the traditional EMT model. Consistent with EpH4 data, we also detect the actin cytoskeleton reorganization and punctate E-cadherin signal in NMuMG mammary epithelial cells upon increased human α-actinin-1 expression without a change in E-cadherin levels (S2F–S2J Fig). Taken together, these results demonstrate that increased α-actinin-1 expression in mammary epithelial cells results in the major reorganization of the actin cytoskeleton and associated E-cadherin-based adhesions without altering E-cadherin levels. This is accompanied by increased cell migration and a loss of polarized acini-like structures. The noted F-actin and E-cadherin phenotype in α-actinin-1-expressing cells is interesting from a cancer perspective, as it resembles dynamic, destabilized cell-cell adhesions described at the margins of collectively migrating epithelial cells [34, 35].

### High α-actinin-1 levels are associated with lack of estrogen receptor expression and poor survival in basal-like breast cancer

**Fig 3:**
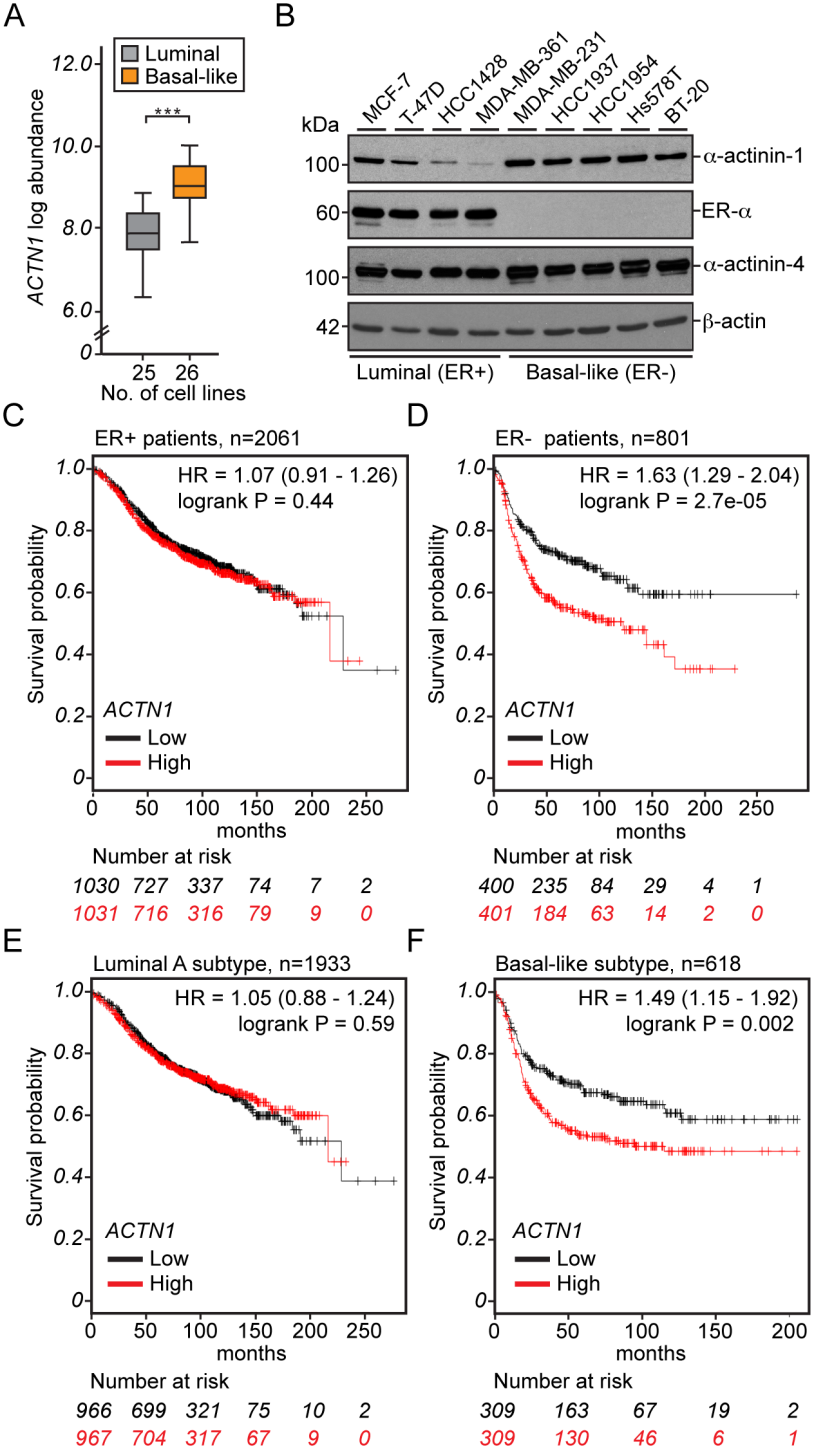
α-actinin-1 expression is higher in basal-like breast cancer cells and is associated with poor survival. (A) Comparison of α-actinin-1 mRNA (*ACTN1* log abundance) between luminal (n = 25) and basal-like (n = 26) breast cancer cell lines. ***P<0.001 by Student’s *t*-test. (B) Western blotting analysis of four luminal and five basal-like breast cancer cell line lysates using α-actinin-1, ER-α, α-actinin-4 or β-actin antibodies as indicated. (C-F) Kaplan-Meier survival analysis showing relapse-free survival (Survival probability) based on α-actinin-1 (*ACTN1*) expression in ER+ (C), ER- (D), luminal A (E) or basal-like (F) breast cancer subtypes. Curves were generated using KM blotter (http://kmplot.com/breast/). Patients with high (red) or low (black) *ACTN1* expression were split based on the median value calculated across the entire dataset to generate two groups of equal size. Numbers of patients at risk at specific time points are indicated below each diagram. n indicates number of patients. Hazard ratios (HRs) and log-rank P-values are depicted for each survival analysis, P-values of <0.05 were considered to be statistically significant.

To further evaluate the significance of increased α-actinin-1 levels in breast cancer, we analysed α-actinin-1 mRNA levels from a gene profiling study of 51 breast cancer cell lines containing 25 luminal (estrogen receptor, ER+) and 26 basal-like (ER-) breast cancer cell lines [36]. This analysis revealed that α-actinin-1 mRNA expression is significantly higher in basal-like cell lines compared to luminal cells (Fig 3A, p-value 6.1x10^-9^). Consistent with the mRNA profiling, α-actinin-1 protein levels are higher in basal-like cells compared to luminal cells (Fig 3B, α-actinin-1). This expression pattern is specific for α-actinin-1, as α-actinin-4 protein levels are relatively constant in all cell lines analysed (Fig 3B, α-actinin-4). These results are interesting, as the basal-like subtype represents an aggressive form of breast cancer, which currently have inadequate treatments [37]. To assess whether the association between higher α-actinin-1 levels and basal-like subtype have a clinical impact, we used a large integrated microarray dataset accompanied with patient survival information [38] (http://kmplot.com/). First, the grouping of breast cancer patients according to their ER status (n = 2862) revealed a striking association between high α-actinin-1 expression and decreased relapse-free survival only in the ER- group. (Fig 3C and 3D, *P* = 2.7x10^5^). Moreover, subsequent analysis using the intrinsic subtype classification [39] indicated that high α-actinin-1 levels decrease the survival probability in the basal-like subtype (Fig 3F), while α-actinin-1 levels lack prognostic value in the luminal subtypes (Fig 3E, luminal A and data not shown). Association analysis lacks the prognostic value also in HER2 (human epidermal growth factor receptor 2) positive and negative patient groups (S3E and S3F Fig). Notably, α-actinin-4 levels do not show any correlation with patient survival probability (S3A and S3B Fig), implying that α-actinin-1 and α-actinin-4 contribute differently to breast cancer progression.

Because our overexpression studies demonstrate that the increased α-actinin-1 levels remodel E-cadherin-based adhesions, we evaluated also whether E-cadherin levels influenced survival. Interestingly, an association between high E-cadherin expression (can also be considered as retained expression) and decreased survival is present in the ER- patient group (S3C and S3D Fig), as well as in the basal-like subtype (data not shown). This observation is interesting in the light of growing evidence that collectively invading and disseminating cancer cells retain E-cadherin expression at cell-cell adhesions [9–11]. Together, these results suggest that basal-like breast cancer patients have poorer prognosis if cancer cells retain E-cadherin expression and have increased α-actinin-1 expression.

### α-actinin-1-cross-linked actin fibers destabilize E-cadherin-based adhesions

**Fig 4:**
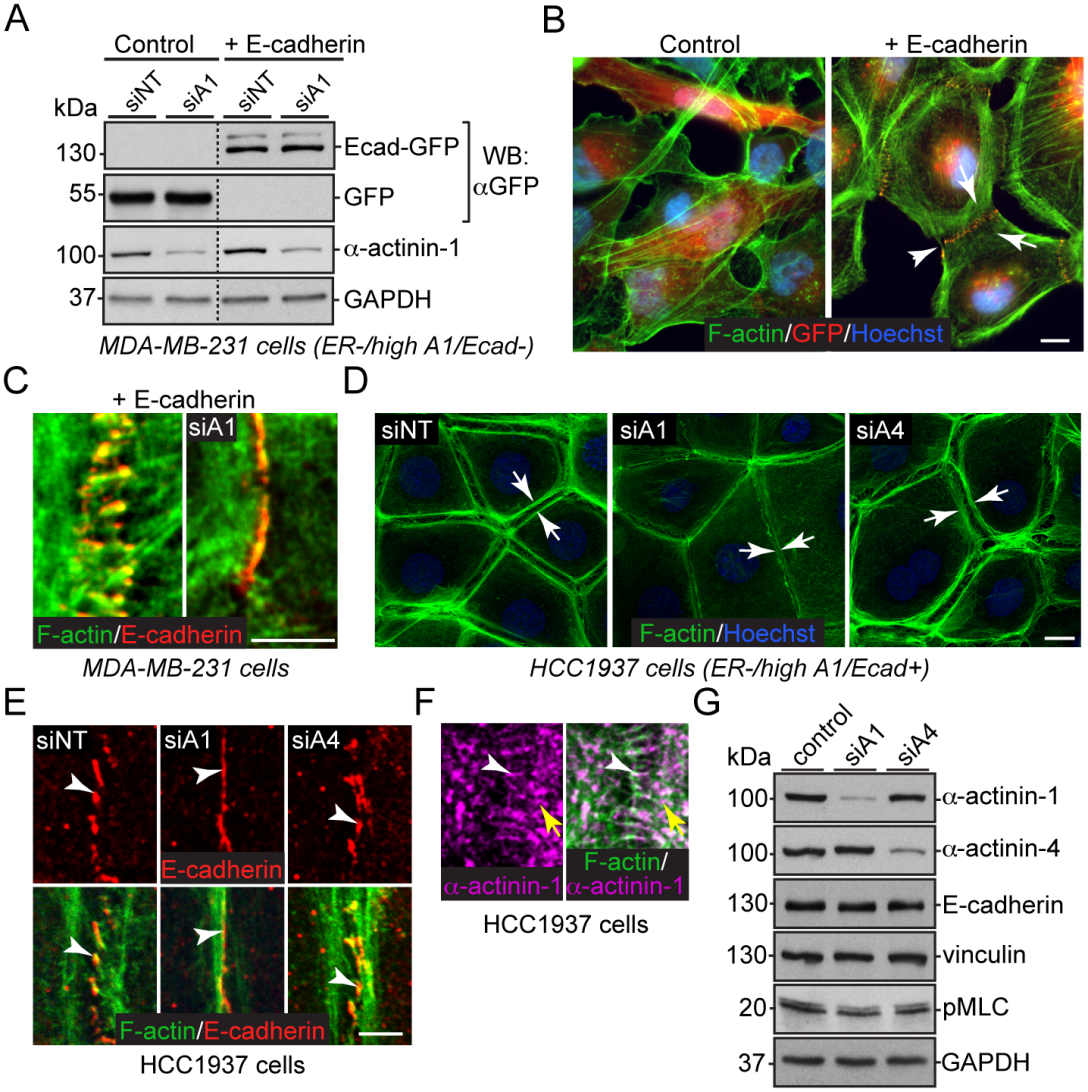
High α-actinin-1 expression in basal-like breast cancer cells destabilizes E-cadherin based adhesions. (A) Western blotting analysis of MDA-MB-231 cells expressing either GFP (Control) or GFP-tagged E-cadherin (+ E-cadherin) in combination with siRNA mediated downregulation using non-targeting (siNT) or α-actinin-1 (siA1) oligos, as indicated. Dotted lines indicate removal of intervening lanes. (B) Merged immunofluorescence images of phalloidin (F-actin, green) stained MDA-MB-231 cells expressing GFP (Control) or GFP-E-cadherin (+ E-cadherin). GFP-signal is pseudo-colored to red, and Hoechst visualizes nuclei. The arrowhead points to punctate E-cadherin and arrows point to subcortical actin fibers. Scale bar, 10 μm (C) A representative example of a change from punctate to linear E-cadherin following α-actinin-1 downregulation (siA1) in MDA-MB-231 cells re-expressing E-cadherin. (D) Phalloidin (F-actin, green) and Hoechst (blue) co-stained HCC1937 cells following siRNA-mediated downregulation using non-targeting (siNT), α-actinin-1 (siA1) or α-actinin-4 (siA4) oligos, as indicated. Arrows show F-actin reorganization. Scale bar, 10 μm. (E) Zoom-in immunofluorescence images of E-cadherin or merged F-actin/E-cadherin following downregulation of control (siNT), α-actinin-1 (siA1) or α-actinin-4 (siA4), as indicated. (F) Immunofluorescence image of a wild-type HCC1937 cell stained for α-actinin-1 (purple) and merged image of α-actinin-1 (purple) and F-actin (green) to demonstrate the localization of endogenous α-actinin-1 on radial (white arrowheads) and arc-like (yellow arrows) actin fibers at E-cadherin based adhesion (see also Fig 5 schematic presentation). (G) Western blotting analysis with the indicated antibodies following downregulation of control (siNT), α-actinin-1 (siA1) or α-actinin-4 (siA4) in HCC1937 cells.

To further study the relationship between increased α-actinin-1 and E-cadherin in basal-like breast cancer cells, we re-expressed GFP (control) or GFP-tagged E-cadherin (+ E-cadherin) in mesenchymal-like MDA-MB-231 cells, which lack ER and E-cadherin expression and have high α-actinin-1 levels (ER-/high A1/Ecad-). Subsequently, we subjected these cells to siRNA-mediated downregulation of control (siNT) or α-actinin-1 (siA1) genes (Fig 4A). Re-expression of E-cadherin in spindle-like MDA-MB-231 cells partially growing on top of each other resulted in the formation of E-cadherin-based cell-cell adhesions (Fig 4B). Notably, these adhesions lack the linear E-cadherin distribution, which is a characteristic of normal epithelial monolayers. Instead, E-cadherin shows a punctate distribution (Fig 4B, arrowhead). This is accompanied by a dramatic reorganization of the actin cytoskeleton, which involves the assembly of subcortical actin fibers that associate with the E-cadherin puncta (Fig 4B, arrows). This phenotype resembles the distribution of E-cadherin and F-actin in non-tumorigenic EpH4 and NMuMG cells following increased α-actinin-1 expression (Fig 2I and S2H Fig). Interestingly, following the downregulation of α-actinin-1 in cells re-expressing E-cadherin, contacts between neighbouring cells frequently merge. In these contacts, the E-cadherin signal becomes linear and aligned with F-actin (Fig 4C). These results demonstrate a direct co-operation between α-actinin-1 levels and the stability of E-cadherin-based adhesions.

To strengthen the MDA-MB-231 overexpression results and to assess whether the noted co-operation is specific for α-actinin-1, we performed siRNA-mediated downregulation of control, α-actinin-1 or α-actinin-4 in basal-like HCC1937 cells. HCC1937 cells lack ER expression and have high α-actinin-1 and endogenous E-cadherin levels (ER-/high A1/Ecad+). F-actin and E-cadherin co-staining revealed that both control and α-actinin-4-downregulated cells form epithelial-like cell-cell contacts, in which punctate E-cadherin associates with subcortical F-actin fibers (Fig 4D and 4E). Consistent with the MDA-MB-231 results, α-actinin-1 downregulation induced the linearization and alignment of F-actin and E-cadherin (Fig 4D and 4E, siA1), providing further evidence that α-actinin-1 regulates the stability of E-cadherin-based adhesions, and indicating that this activity is specific to α-actinin-1.

To assess the distribution of α-actinin-1 in HCC1937 cells that have punctate E-cadherin-based adhesions, we stained for α-actinin-1 to assess its distribution. In contrast to non-tumorigenic mammary epithelial cells, which primarily have cytoplasmic and faint cell-cell contact staining of α-actinin-1 (S2A Fig), the α-actinin-1 staining in HCC1937 cells shows a prominent dotted-like signal on short F-actin fibers originating from E-cadherin puncta (Fig 4F, white arrowhead, radial fibers), as well as along a subcortical F-actin network (Fig 4F, yellow arrow, arc-like fibers). This staining pattern implies that α-actinin-1 crosslinks E-cadherin-associated actin fibers.

**Fig 5:**
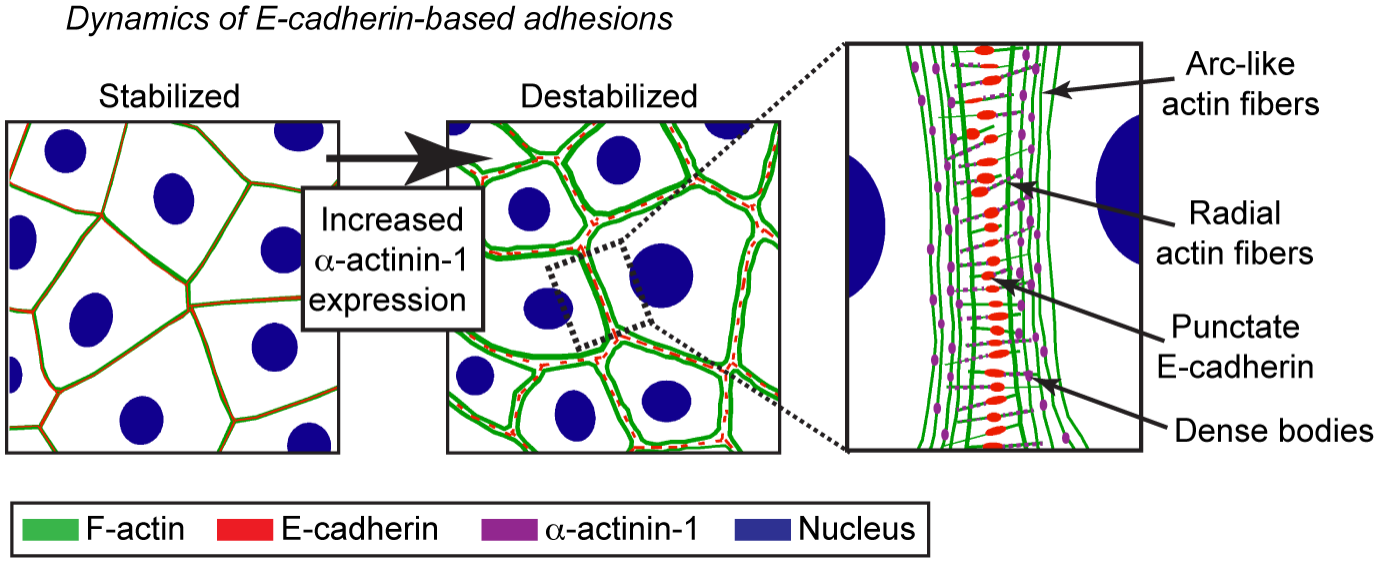
Schematic presentation of destabilized E-cadherin-based adhesions and associated actin fibers following increased α-actinin-1 expression.

In previous studies, loss of E-cadherin stability has been associated with increased tension at adhesions. This has been assayed through the recruitment of vinculin to E-cadherin-based adhesions, as well as an increase in phosphorylated myosin light chain (pMLC) at cell-cell contacts [12, 35, 40]. Co-staining of vinculin and pMLC revealed that downregulation of α-actinin-1 only partially decreased the signal intensity compared to control or α-actinin-4-downregulated cells, although both the vinculin and pMLC distribution pattern was altered (S4A Fig). Consistent with these results, vinculin levels remained unaltered, and pMLC levels were sometimes slightly decreased in both α-actinin-1 and α-actinin-4 downregulated cells (Fig 4G). Taken together, these results demonstrate that in the presence of E-cadherin, increased α-actinin-1 levels induce the assembly of E-cadherin-associated actin fibers that destabilize cell-cell contacts (Fig 5). This partially occurs through increased tension at adhesions.

## Discussion

In the present study, we found that α-actinin-1 expression is increased in breast cancer and is associated with decreased survival in patients with basal-like subtype. In breast cancer cells, increased α-actinin-1 reorganizes the actin cytoskeleton, resulting in the destabilization of E-cadherin-based adhesions without affecting E-cadherin expression levels. These results demonstrate that increased α-actinin-1 expression can contribute to cancer progression through partial EMT. Partial EMT (also called intermediate, hybrid EMT) is highly relevant, as growing evidence has suggested that a significant portion of cancer cells collectively escape from primary tumors instead of the traditional EMT model, where single cells escape [2, 7, 8, 41]. Collective cell escape has been considered to be advantageous for metastasis because these cell clusters can be more resistant to apoptosis and have more tumor-initiating potential compared to single migrating cells [6, 42]. Interestingly, the resembling E-cadherin-actin organization phenotype, which we observed following increased α-actinin-1 expression, has been previously described in transformed epithelial cells upon Ras mutation or chemical carcinogens [43], and considered to reflect a partial EMT process [44]. Additionally, it is worth noting that a loss of the polarity protein PAR3 [45] and the F-actin stabilizer EPLIN [34] results in punctate E-cadherin distribution and compromised cell-cell adhesions. In future studies, it will be important to evaluate whether these molecules regulate the dynamics of E-cadherin-based adhesion in co-operation with α-actinin-1 or in more specific ways. Another noteworthy point in future studies is to address a possible cross-talk between E-cadherin and integrin based adhesions, since in addition to α-actinin-1 e.g. Ras and EPLIN function at both adhesions.

Considering the underlying mechanisms that cause punctate E-cadherin distribution in α-actinin-1 high cells, elevated tension through newly assembled actin fibers is an obvious candidate. In the E-cadherin complex, α-catenin functions as a mechanosensitive protein, which recruits vinculin through a force-dependent conformational change of α-catenin [35, 40]. This conformational change can be assayed by increased vinculin intensity at E-cadherin-based adhesions, and it is considered to strengthen the linkage between E-cadherin and actin fibers. Other lines of studies have demonstrated that α-actinin-1 can interact with both vinculin [46] and α-catenin [47]. Although we detect changes in vinculin distribution following the downregulation of α-actinin-1, we do not observe a dramatic decrease in signal intensity at adhesions. Consistent with this, myosin light chain phosphorylation, which is indicative of myosin II activity, showed only a minor decrease. These results suggest that although tension takes part in remodeling E-cadherin-based adhesions to form the punctate pattern observed in α-actinin-1 high cells, additional mechanisms are also likely to be involved. It is worth noting that similar to integrin-based adhesions, E-cadherin adhesions can act as templates for actin polymerization, resulting in straight, radial actin fibers [43, 48], which we detected in cells expressing high levels of α-actinin-1. In mesenchymal cells, the assembly of radial fibers (or dorsal stress fibers) from integrin-based adhesions at leading lamella requires α-actinin-1 and Rac1 [32, 49, 50], suggesting that a similar mechanism might be active at E-cadherin adhesions. Upon downregulation of α-actinin-1, punctate E-cadherin contacts merge to form a linear contact due to a lack of active polymerization of radial fibers. In epithelial cells, several formin family members of actin nucleators, such as mDIA1, FMNL3, and FMLN2, are involved in the assembly of actin fibers originating from adherens junctions [51, 52]. FMNL2 is of particular interest for future studies, as it is required downstream of Rac1 for the assembly of immature cell-cell contacts [51]. A third possible remodeling mechanism is a role for dense bodies along actin fibers, regions where α-actinin-1 cross-links F-actin. The analogous structure in muscle cells, called Z-discs, function as important signaling centers that regulate muscle cell adhesion stability [53]. In addition to their cross-linking functions, muscle-specific α-actinins act as scaffold proteins. Therefore, an interesting possibility in α-actinin-1 high cells is that α-actinin-1 at dense bodies along the reorganised actin fibers function as signalling center in the vicinity of E-cadherin. A recent study that mapped protein organization in E-cadherin-based adhesions by super-resolution microscopy identified Abl kinase as an interesting candidate signalling molecule [73], which regulates E-cadherin dynamics [54] and has been shown to associate with α-actinin-1 at dense bodies through ArgBP2 (Abl/Arg kinase adapter) [55].

From a clinical standpoint, our main finding is that high α-actinin-1 levels are associated with decreased relapse-free survival in basal-like breast cancer. This association is interesting, as the basal-like subtype represents an aggressive form of breast cancer with limited therapy response [37]. The limited response is partly due to the lack of ER receptor, which precludes the therapeutic use of hormone-based interventions. Furthermore, a portion of basal-like tumors, triple-negative breast cancers, lacks the therapeutic target HER2 (human epidermal growth factor receptor 2). With regards to triple-negative breast cancer, α-actinin-1 levels are not associated with survival prognosis, although some caution should be taken due to the small sample number compared to the other subtypes examined (data not shown). These observations suggest that the association between high α-actinin-1 expression and decreased survival is restricted to the basal-like subtype independent of HER2 status. This is interesting in light of gene expression profiling studies proposing that the current molecular classification for breast cancer based on the relative expression of approximately 500 genes, dubbed as the “intrinsic” set [37, 56] requires additional subclassification [57–59].

Our breast cancer cell line analysis provides additional evidence for the association between high α-actinin-1 and the basal-like subtype. This relationship is specific for α-actinin-1, as both mRNA and protein analyses indicate that α-actinin-4 levels do not show major alterations between basal-like and luminal breast cancer cells. While it is clear that gene amplification accounts for a portion of the reported upregulation of α-actinin-4 in various cancers [17, 18, 60], neither cancer database searches (http://www.cbioportal.org) nor copy-number variations in breast cancer cell lines [36] supports gene amplification as an underlying mechanism for the increased α-actinin-1 expression. Considering alternative mechanisms for α-actinin-1, previous studies have reported that transforming growth factor beta, TGF-β, induces α-actinin-1 mRNA levels [61, 62]. In agreement with this, we detect increase in α-actinin-1 protein levels both in EpH4 and NMuMG cells following TGF-β treatment (S4B Fig). In breast tissue, TGF-β has a dual role. In healthy breast tissue, it functions as a tumor suppressor, but during cancer progression, it can act as a tumor promoter [63]. Interestingly, this switch is thought to be associated with ER activity [64]. Under healthy conditions, ER activation inhibits the transcriptional activity of TGF-β, and it inhibits TGF-β-induced migration. However, in cancer tissues, the TGF-β response signature correlates with ER-negative tumors and poor prognosis [65–67]. Therefore, it is will be interesting to study the activation of TGF-β and increased α-actinin-1 expression in basal-like (ER-) breast cancer further.

In conclusion, our results significantly broaden the current understanding of α-actinin-1 in cancer cells [68, 69]. The expression changes of α-actinin-1 are not limited to breast cancer based on gene expression profiling screens [26–28, 70] and our results, and therefore warrant additional studies in other tissues. Improved understanding of the molecular basis of the basal-like subtype and its pathophysiology is important to identify and develop prognostic markers and therapy options. The present study proposes to explore the value of α-actinin-1 as a prognostic biomarker in basal-like breast cancer.

## Materials and methods

### Cell culture, antibodies and reagents

All cell lines were purchased from American Type Culture Collection (ATCC) and cultured according to protocol provide by ATCC at 37°C in 5% CO_2_. Following primary rabbit polyclonal antibodies were used: anti-α-actinin-1 (A1-A341 [32]), anti-α-actinin-4 (ALX-210-356, Alexis Biochemicals), anti-laminin (L9393, Sigma-Aldrich), anti-GFP (ab290, Abcam), anti-pMLC (#3671, Cell Signaling), anti-E-cadherin (24E10, #3195, Cell Signaling). Primary mouse monoclonal antibodies were: anti-E-cadherin (HECD-1, ab1416, Abcam), anti-ER-α antibody (sc-8002, Santa Cruz Biotechnology), anti-vinculin (V9131, Sigma-Aldrich). In addition the used primary antibodies were: rat monoclonal anti-E-cadherin antibody (U3254, Sigma-Aldrich), rabbit monoclonal anti-GAPDH (2118S, Cell Signaling), mouse monoclonal anti-β-actin antibody (A1978, Sigma). Secondary antibodies in western blotting were anti-rabbit-HRP and anti-mouse-HRP (Chemicon International) and in immunofluorescence Alexa Fluor^®^ anti-rabbit, anti-mouse or anti-rat (Invitrogen). Filamentous actin was stained with Alexa Fluor^®^ 488, 546 or 647 phalloidin (Invitrogen).

### *In silico* data mining

*ACTN1* gene expression profiling across healthy and cancerous tissues was performed with the MediSapiens database [29]. For Kaplan-Meier curves a correlation between gene expression and patient relapse free survival was studied by splitting *ACTN1* (probe 208636_at) expression into two groups according to median (low and high). Statistical significance is based on logrank P value (http://kmplot.com/) [38, 39].

### Immunohistochemistry, quantification and analysis of A1-341 antibody specificity

Paraffin-embedded breast cancer tissue microarrays (BRM961, BRC481, BRC482 and BRC483) were purchased from US Pantomics, Inc (California, USA). All patients were female, average age was 42 (29–72), and age did not correlate with α-actinin-1 expression. 87% of samples were invasive ductal carcinoma, 8,7% invasive lobular carcinoma and 4,3% ductal carcinoma in situ. Immunohistochemistry was performed according to standard protocols after heat induced antigen retrieval (S1699, Dako) and peroxidase-blocking (S202386-2, Dako). Primary antibody was incubated overnight at 4°C. Streptavidin-HRP (P039701-2, Dako) based DAB chromogen (K346811-2, Dako) was used for detection together with hematoxylin staining. The stained arrays were imaged with Pannoramic 250 Flash II digital slide scanner and viewed with Pannoramic viewer (3DHISTECH Ltd) at Genome Biology Unit (Faculty of Medicine, and HiLIFE at University of Helsinki and Biocenter Finland). DAB signal was quantified with ImageJ H DAB vector plugin software by generating a D-HSCORE value described in [33]. To exclude fat cells from healthy breast tissue sections, regions of interest (ROIs) were selected to cover healthy ductal area, and similar size of ROIs were used for cancer tissue analysis (on average three per sample). To study an applicability of the rabbit polyclonal A1-341 α-actinin-1 antibody [32] in immunohistochemistry, control or α-actinin-1 silenced EpH4 cells were fixed with 4% paraformaldehyde, embedded in 5% agarose and in paraffin and sectioned prior to immunohistochemistry. In addition, a specific (targeting the A1-341 epitope DHYDSQQTND) or a nonspecific synthesized peptide blocking was used to test antibody specificity in breast cancer sections. The antibody-peptide mix was incubated for 1 hour prior to adding the primary antibody according to immunohistochemistry protocol.

### Protein microarray patient lysates and quantification

SomaPlex Breast Cancer Tissue Lysate Protein Microarray (PMA2-001-L, Protein Biotechnologies) contained paired healthy and cancer tissue, of which 91% were ductal, 2% lobular and 7% other breast carcinoma subtypes. The majority was scored as advanced grade 2 or 3 (85%) and average age was 50,5 (32–85), and the age did not correlate with α-actinin-1 expression. Tissues were lysed in RIPA buffer at equal concentrations and transferred in triplicates on a PVDF membrane. Western blotting analysis and ECL (SuperSignal West Femto, PIERCE) were performed according to standard protocol. Images were acquired with ChemiDoc^™^ XRS System (Bio-Rad) equipped with Image Lab^™^ Software and quantified using ImageStudioLite software (Odyssey; LI-COR Biosciences).

### Overexpression and siRNA mediated gene downregulation

Open reading frame of human *ACTN1* or GFP in pENTR223 entry vector were transferred into the pcDNA6.2/N-EmGFP-DEST destination vector (Invitrogen) to generate GFP-tagged α-actinin-1 and GFP control at Genome Biology Unit core facility (University of Helsinki). Human CDH1-pcDNA3 plasmid was obtained from Addgene [71]. To generate stable EpH4, NMuMG or MDA-MB-231 cells expressing GFP (control) or GFP-tagged α-actinin-1 (α-actinin-1) or GFP-tagged E-cadherin (+ E-cadherin) cells were transfected using Lipofectamine2000 according to provided protocol, and cultured under blasticidin (15205, Sigma-Aldrich) or geneticin (G418, 10131019, Invitrogen) selection.

For siRNA mediated downregulation of *ACTN1* or *ACTN4* mix of two oligos were used for each gene: *ACTN1*; J-011195-05, J-011195-06 and A*CTN4*; J-011988-08, J-011988-09 (Dharmacon). For control non-targeting siRNA oligo was D-001206-13 (Dharmacon). To improve downregulation efficiency Lipofectamine2000 (Invitrogen) mediated transfection was conducted on two consecutive days following the a day after seeding, and samples for western blotting and immunofluorescence analyses were collected 72–82 hours after the first transfection.

### Wound healing, three dimensional Matrigel cultures and TGF-β assay

For the wound healing assay equal amount of EpH4 or HCC1937 cells from each line were seeded on 6-well plates 24–48 hours before generating the wounds with a sterile tip to a confluent monolayer. With EpH4 cells 24-hours time-lapse phase-contrast imaging was performed by taking images every hour using Cell-IQ (CM Technologies) platform equipped with 10x objective and 37°C in 5% CO_2_ incubator. HCC1937 cells were imaged at beginning of the wound and at the end 20-hour time point. Images were analyzed and quantified by ImageJ software 1.51 by calculating open areas in pixels. QuickTime Player Pro 7.6 was used for generating movies.

For three-dimensional Matrigel cultures previously described method was used [72]. Briefly, control and α-actinin-1 expressing EpH4 lines were plated on 8-well chamber polystyrene vessel tissue culture treated glass slides (cat no. 354108, Corning) coated with phenol red-free Matrigel matrix (BD Bioscience). Cell media was supplemented with 250μg/ml prolactin (cyt-267, ProSpec), 1μg/ml hydrocortisone (Sigma-Aldrich), 5μg/ml hypurin bovine neutral insulin (Tamro) and 2–4% Matrigel. Cells were cultured for seven to ten days and media was changed every third day.

In TGF-β experiments EpH4 or NMuMG cells were serum starved with 0,1% BSA cell culture media for 24 h prior to treatment with or 5 ng/ml TGF-β1 or left untreated (control) for 24 h.

### Immunofluorescence and western blotting analysis

For immunofluorescence analysis cells seeded on glass coverslips were fixed with 3.5% (w/v) paraformaldehyde (PFA) for 10–15 minutes and washed three times with TBS prior to permeabilization with 0.1% Triton X-100 for 5 minutes followed by three TBS washes. For blocking 5% goat serum in TBS for 30 minutes was used prior to adding a primary antibody for 30–60 minutes, followed by three TBS washes before labeled with secondary antibody for 30 minutes, and washed three times with TBS. Before mounting (Immumount, Thermo Scientific) cells were labeled also with Hoechst. Immunofluorescence for three-dimensional Matrigel cultures was conducted based on available protocols from professor Joan S. Brugge laboratory (https://brugge.med.harvard.edu/protocols). Stained coverslips were analyzed and imaged using upright Zeiss AxioImager M2 epifluorescence microscope equipped with 20x/Plan-Apochromat/0.8/M27, 63x/Plan-Apochromat/1.40 Oil/M27 and AxioCam HRm camera or inverted Leica TCS CARS SP8 laser scanning confocal microscope equipped with 63x/HC PL APO CS2/1.20 water objective. Final images were generated using Adobe photoshop and Illustrator CS6.

For western blotting cells were lysed using SDS boiling buffer (2.5% SDS, 0.25 M Trizma base), needled (25-gauge) and centrifuged at 14 800 rpm for 10 minutes at 4°C prior to protein concentration measurements using Bio-Rad DC protein assay (Bio-Rad Laboratories). 10–30 μg of lysates were run on SDS-PAGE gel, and for western blotting analysis TBS including 0.05% Tween 20 and 5% w/v nonfat dry milk or BSA were used for blocking and antibody incubations. Primary antibodies were incubated overnight at 4°C and secondary antibodies for 30 minutes prior to detection with ECL reagents (SuperSignal West Femto, PIERCE).

### Image quantification and statistical analysis

For quantification of F-actin intensity, image stacks were generated in ImageJ software 1.48v (National Institutes of Health, USA), and borders of individual cells were marked using E-cadherin staining. F-actin intensity of each cell was quantified with integrated density (Int Den) tool. Values were normalized to control cells. Acini-like structures were quantified from phase-contrast images by drawing lines around each structure, and subsequently area and circularity of those structures were measured using ImageJ software 1.48v (National Institutes of Health, USA). Statistical analyses were performed using Student’s *t*-test, assuming a two-tailed distribution and unequal variance.

## Supporting information

S1 Fig The rabbit polyclonal A1-341 antibody is specific for α-actinin-1. (A) Immunohistochemistry images of paraffin-embedded control (NT-KD) or α-actinin-1 (A1-KD) downregulated EpH4 mammary epithelial cells stained with the rabbit polyclonal α-actinin-1 A1-341 antibody [32]. (B) Western blotting analysis with same antibody to show knockdown efficiency. GAPDH was used as a loading control. (C) Immunohistochemistry images of paraffin-embedded breast cancer tissue sections stained for the A1-341 antibody, which was pre-incubated with a nonspecific peptide (Nonspecific) or with a specific peptide (Specific blocking) prior to staining. (D) An example of immunohistochemistry image of paraffin-embedded healthy and cancer breast tissue stained for the A1-341 antibody illustrating the region of interest (ROI) used in the D-HSCORE analysis. Scale bar, 50 μm.
(TIF)

S2 Fig Stable ectopic expression of α-actinin-1 in EpH4 and NMuMG mammary epithelial cells. (A) Immunofluorescence images stained for α-actinin-1 antibody A1-341 (top panel: red) and phalloidin (lower panel: F-actin, green) of EpH4 cells stably expressing GFP (Control) or GFP-tagged α-actinin-1 (α-actinin-1). Hoechst is included to visualize nuclei. Arrows show α-actinin-1 localization on actin fibers. Scale bar, 10 μm. (B, F, G) Western blotting analysis with the indicated antibodies from the selected stable EpH4 (B) and NMuMG (F,G) control and α-actinin-1 lines (#1, #2). Dotted lines indicate removal of intervening lanes. (C) Phase-contrast images of acini-like structures from control and α-actinin-1 expressing cells that were grown on three-dimensional Matrigel gel (3D Matrigel culture) for seven days. (D) Quantification (n = 68-87/line #) of area and circularity of acini-like structures shown in (C). Arbitrary area values are normalized to control cells. Scale bar, 50 μm. (E) Merged immunofluorescence images of laminin (green) and Hoechst (blue) stained control and α-actinin-1 expressing EpH4 cells grown on Matrigel for seven days. Scale bar, 20 μm. (H) Control and α-actinin-1 expressing NMuMG cells stained for F-actin (green) and Hoechst (blue). Arrows indicate the reorganization of F-actin. Scale bar, 10 μm. (I) Quantification (n = 45-65/line #) of F-actin intensity shown in (H) from two independent experiments. Arbitrary values are normalized to control cells. Error bars indicate s.d. ***P<0,001 by Student’s *t*-test. (J) Merged immunofluorescence images of phalloidin (F-actin, green), E-cadherin (red) and Hoechst (blue) of control or α-actinin-1 NMuMG cells. Arrowheads indicate the E-cadherin pattern. Insets are zoomed-in to show E-cadherin and F-actin staining alone. Scale bar, 10 μm.
(TIF)

S3 Fig Survival analysis for α-actinin-4 and E-cadherin. Kaplan-Meier survival analysis showing relapse free survival (Survival probability) based on the expression of α-actinin-4 (*ACTN4*), E-cadherin (*CDH1*) or α-actinin-1 (*ACTN1*) in ER+ (A,C) or ER- (B,D) or HER2+ (E) or HER- (F) breast cancer subtypes as indicated. Curves were generated using KM blotter (http://kmplot.com/breast/). Patients with high (red) or low (black) *ACTN4*, *CDH1 or ACTN1* expression are split based on the median value calculated across the entire dataset to generate two groups of equal size. Numbers of patients at risk at specific time points are indicated below each diagram. Sample size is indicated above each diagram. Hazard ratios (HR) and log-rank P-values are depicted for each survival analysis. P-values of < 0.05 were considered to be statistically significant.
(TIF)

S4 Fig Reorganization of vinculin and pMLC following downregulation of α-actinin-1 in HCC1937 cells, and TGF-β induces α-actinin-1 protein expression. (A) Phalloidin (F-actin, green), vinculin (white) and pMLC stained (red) co-staining HCC1937 cells following siRNA mediated downregulation using non-targeting (siNT), α-actinin-1 (siA1) or α-actinin-4 (siA4) oligos as indicated. Arrowheads show vinculin and pMLC reorganization in α-actinin-1 downregulated cells. Scale bar 10 μm. (B) Western blotting analysis to show that 24 h TGF-β treatment induces α-actinin-1 protein expression without changing E-cadherin levels both in EpH4 and NMuMG cells. GAPDH is a loading control.
(TIF)

S1 Movie 24-hour time-lapse imaging every hour after scratch wounding of control and α-actinin-1-expressing EpH4 cells. (MOV)
