# Supplementary figures and images for: Increased α-actinin-1 destabilizes E-cadherin-based adhesions and associates with poor prognosis in basal-like breast cancer
Source: PLoS One. 2018 May 9;13(5):e0196986. doi: 10.1371/journal.pone.0196986 (PMC5942811; doi:10.1371/journal.pone.0196986)

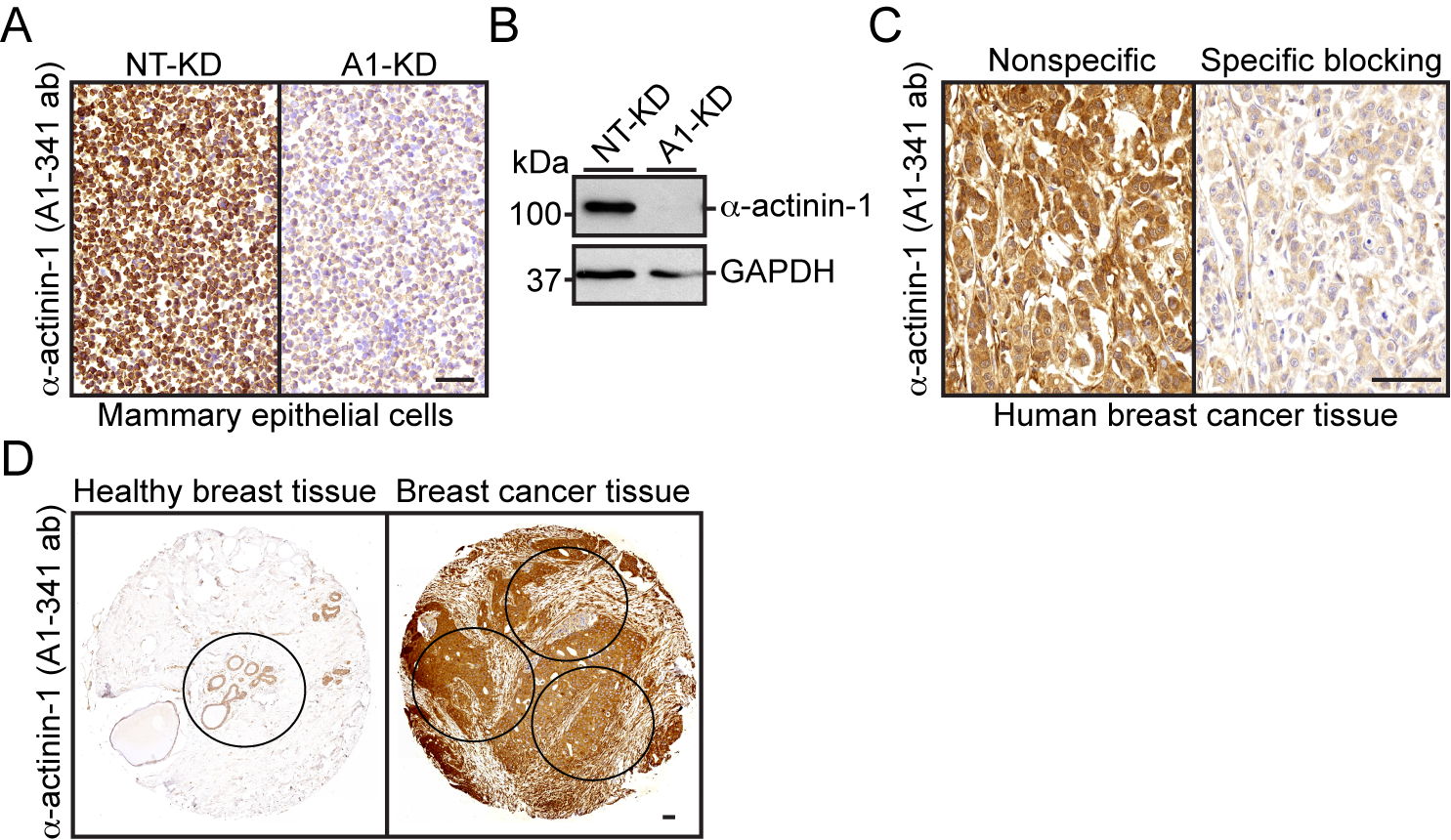

Supplement: S1 Fig — (A) Immunohistochemistry images of paraffin-embedded control (NT-KD) or α-actinin-1 (A1-KD) downregulated EpH4 mammary epithelial cells stained with the rabbit polyclonal α-actinin-1 A1-341 antibody [32]. (B) Western blotting analysis with same antibody to show knockdown efficiency. GAPDH was used as a loading control. (C) Immunohistochemistry images of paraffin-embedded breast cancer tissue sections stained for the A1-341 antibody, which was pre-incubated with a nonspecific peptide (Nonspecific) or with a specific peptide (Specific blocking) prior to staining. (D) An example of immunohistochemistry image of paraffin-embedded healthy and cancer breast tissue stained for the A1-341 antibody illustrating the region of interest (ROI) used in the D-HSCORE analysis. Scale bar, 50 μm. (TIF) [file pone.0196986.s001.tif]

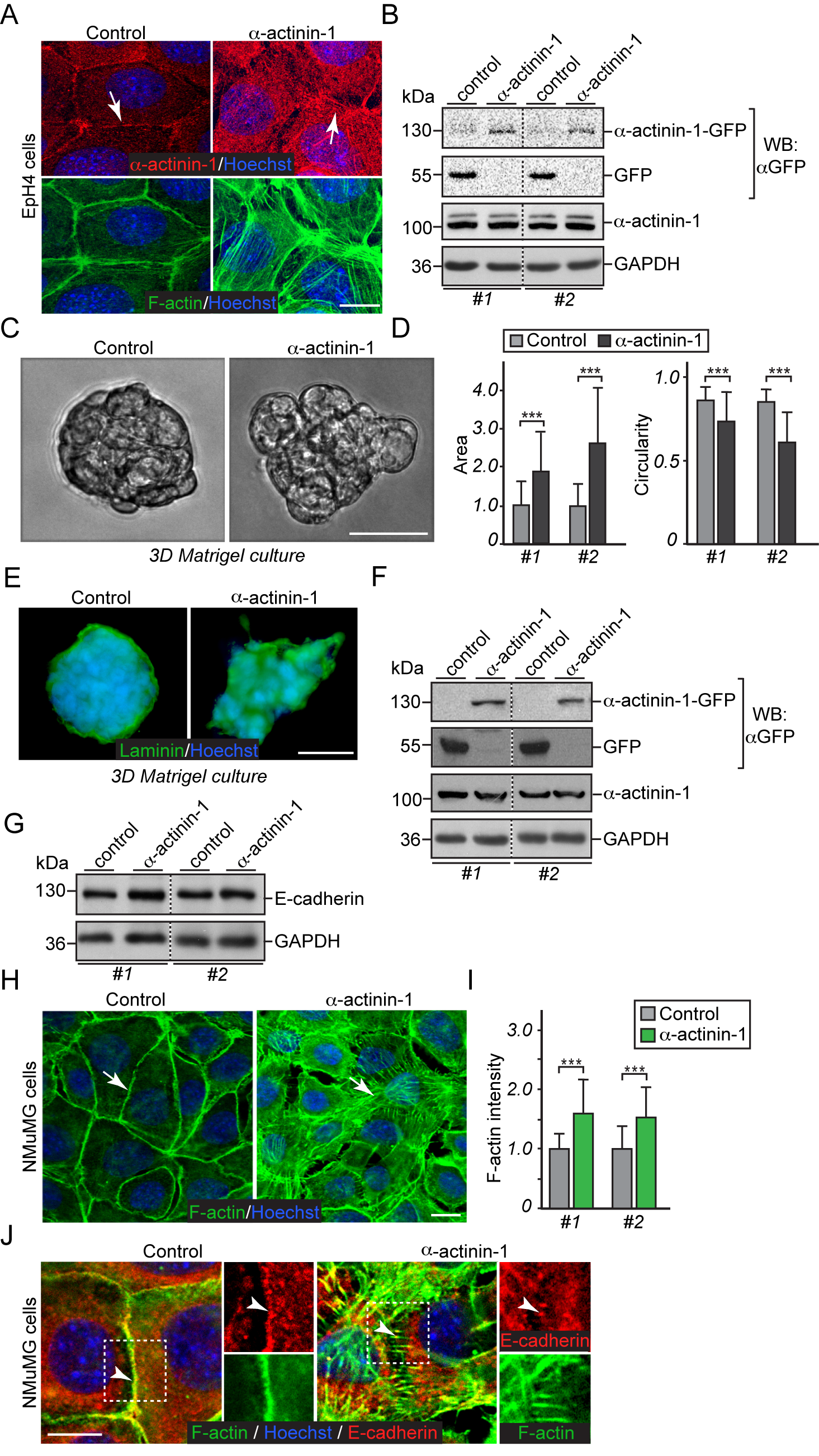

Supplement: S2 Fig — (A) Immunofluorescence images stained for α-actinin-1 antibody A1-341 (top panel: red) and phalloidin (lower panel: F-actin, green) of EpH4 cells stably expressing GFP (Control) or GFP-tagged α-actinin-1 (α-actinin-1). Hoechst is included to visualize nuclei. Arrows show α-actinin-1 localization on actin fibers. Scale bar, 10 μm. (B, F, G) Western blotting analysis with the indicated antibodies from the selected stable EpH4 (B) and NMuMG (F,G) control and α-actinin-1 lines (#1, #2). Dotted lines indicate removal of intervening lanes. (C) Phase-contrast images of acini-like structures from control and α-actinin-1 expressing cells that were grown on three-dimensional Matrigel gel (3D Matrigel culture) for seven days. (D) Quantification (n = 68-87/line #) of area and circularity of acini-like structures shown in (C). Arbitrary area values are normalized to control cells. Scale bar, 50 μm. (E) Merged immunofluorescence images of laminin (green) and Hoechst (blue) stained control and α-actinin-1 expressing EpH4 cells grown on Matrigel for seven days. Scale bar, 20 μm. (H) Control and α-actinin-1 expressing NMuMG cells stained for F-actin (green) and Hoechst (blue). Arrows indicate the reorganization of F-actin. Scale bar, 10 μm. (I) Quantification (n = 45-65/line #) of F-actin intensity shown in (H) from two independent experiments. Arbitrary values are normalized to control cells. Error bars indicate s.d. ***P<0,001 by Student’s t-test. (J) Merged immunofluorescence images of phalloidin (F-actin, green), E-cadherin (red) and Hoechst (blue) of control or α-actinin-1 NMuMG cells. Arrowheads indicate the E-cadherin pattern. Insets are zoomed-in to show E-cadherin and F-actin staining alone. Scale bar, 10 μm. (TIF) [file pone.0196986.s002.tif]

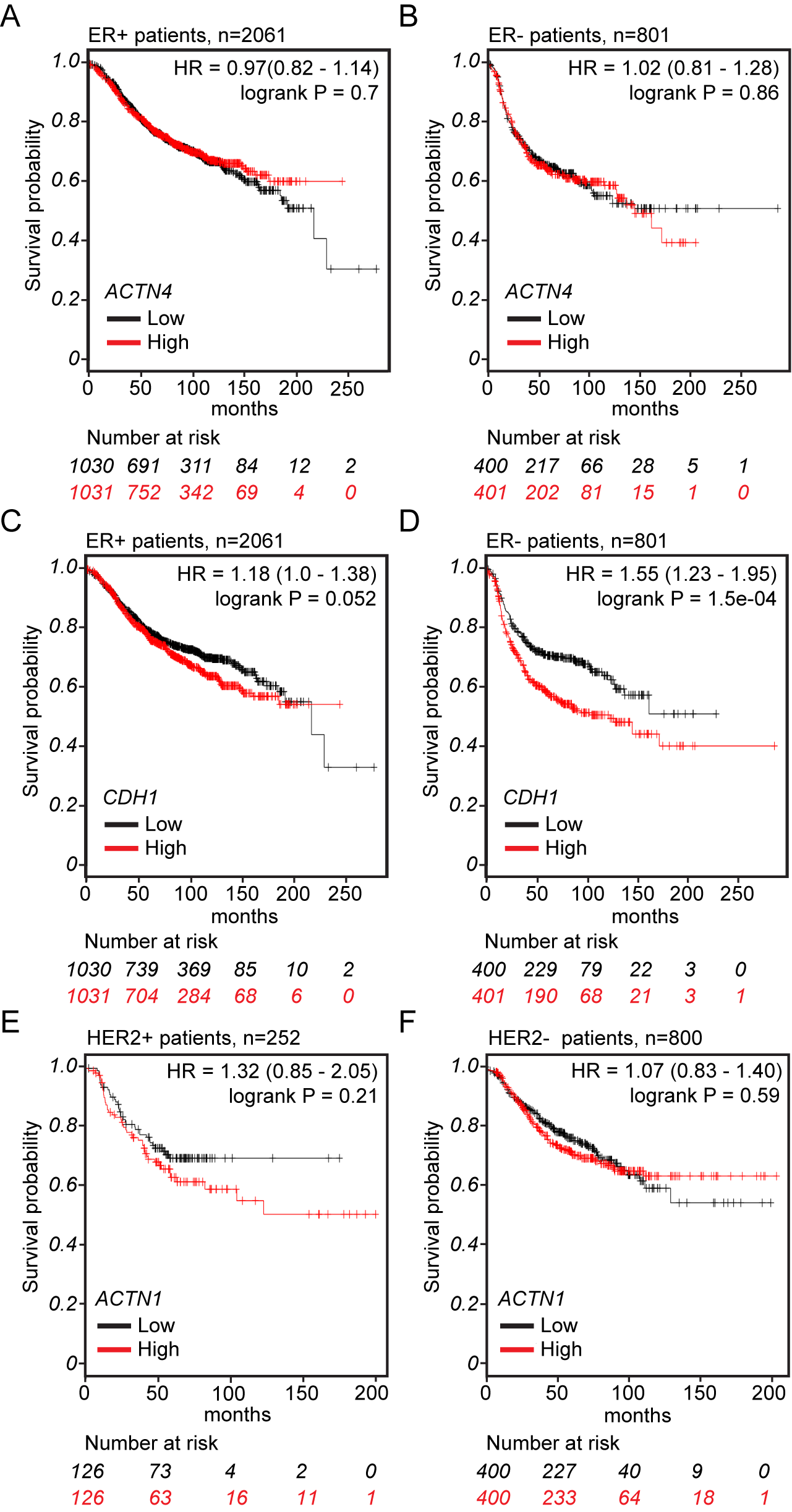

Supplement: S3 Fig — Kaplan-Meier survival analysis showing relapse free survival (Survival probability) based on the expression of α-actinin-4 (ACTN4), E-cadherin (CDH1) or α-actinin-1 (ACTN1) in ER+ (A,C) or ER- (B,D) or HER2+ (E) or HER- (F) breast cancer subtypes as indicated. Curves were generated using KM blotter (http://kmplot.com/breast/). Patients with high (red) or low (black) ACTN4, CDH1 or ACTN1 expression are split based on the median value calculated across the entire dataset to generate two groups of equal size. Numbers of patients at risk at specific time points are indicated below each diagram. Sample size is indicated above each diagram. Hazard ratios (HR) and log-rank P-values are depicted for each survival analysis. P-values of < 0.05 were considered to be statistically significant. (TIF) [file pone.0196986.s003.tif]

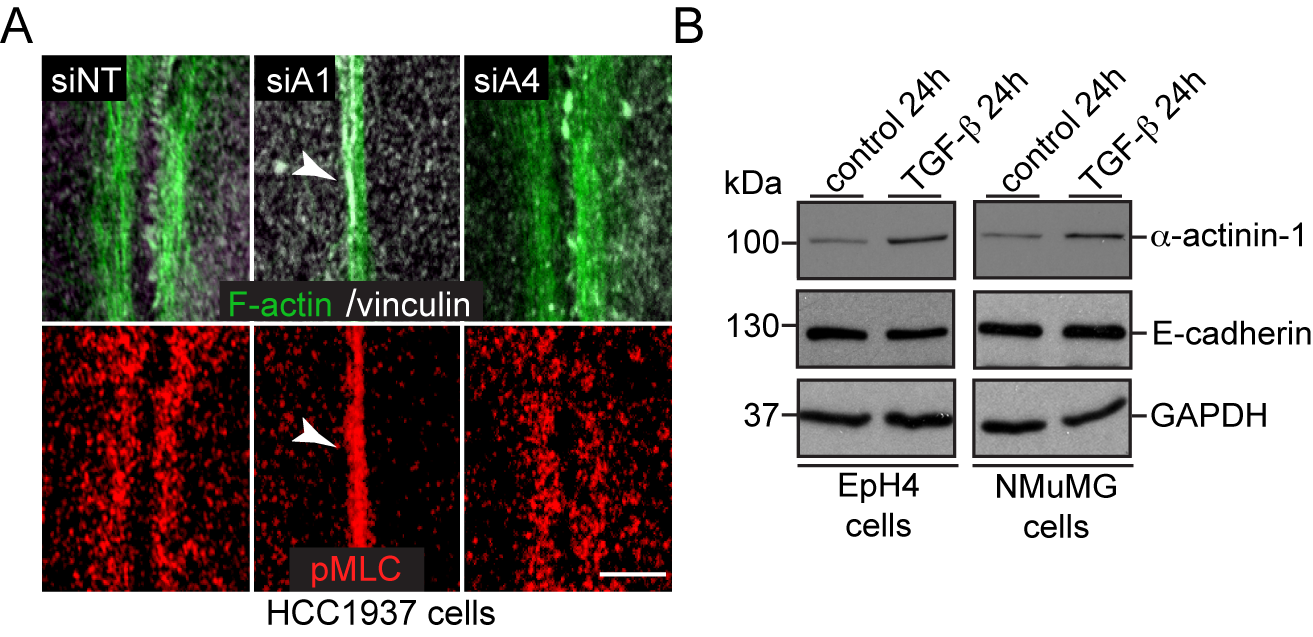

Supplement: S4 Fig — (A) Phalloidin (F-actin, green), vinculin (white) and pMLC stained (red) co-staining HCC1937 cells following siRNA mediated downregulation using non-targeting (siNT), α-actinin-1 (siA1) or α-actinin-4 (siA4) oligos as indicated. Arrowheads show vinculin and pMLC reorganization in α-actinin-1 downregulated cells. Scale bar 10 μm. (B) Western blotting analysis to show that 24 h TGF-β treatment induces α-actinin-1 protein expression without changing E-cadherin levels both in EpH4 and NMuMG cells. GAPDH is a loading control. (TIF) [file pone.0196986.s004.tif]
